# Books and Magazines

**Published:** 1910-04

**Authors:** 


### Books and Magazines.

SERUM DIAGNOSIS OF SYPHILIS AND BUTYRIC ACID TEST FOR SYPHILIS. By Heideyo Noguchi, M. D., M. Sc., Associate Member of the Rockefeller Institute for Medical Research, N. Y. Fourteen illustrations. Philadelphia and London: J. B. Lippincott Co. 1910. Cloth, price, $2.00.

"The object of this book is, first, to give a brief yet adequate account of the principles of serum hæmolysis and the behaviors of the combinations of antigens and antibodies towards hæmolysis, so essential for a proper understanding of the subject; discussing at some length the quantitative relationship of the factors playing a part in these phenomena, an aspect of the subject that has not perhaps received the consideration that it deserves; and, secondly, to give in detail the technic of Wasserman's method and of the method recommended by the author," so says the author, in his preface, and I am sure I could not say it better. If the book contained nothing else except the technic of Wasserman's method of diagnosis it would be worth more than the price ($2.00) for not one doctor out of a hundred who will read this knows anything of it and of course does not appreciate its great value as a reliable method of diagnosis.

---

POKER-JIM, GENTLEMAN; A CALIFORNIA STORY, and Other Tales and Sketches. By G. Frank Lydston, M. D. The Monarch Book Co., Publishers, Chicago. Illustrated. Handsome cloth covers, illuminated. 396 pages; price, $1.50.

This book written in Dr. Lydston's characteristic and inimitable "stuccato" style is just charming; no, it is fascinating, if there is any difference in the meaning of the words. It holds you like a live wire; you can't let go, but it does not burn your hands; it just thrills you. This is the second edition. It deals with the romance of the early days of the mining fever in California when every fellow toted a gun, of course. Jim belonged to the John Oakhurst class. Dr. Lydston dedicates the book to the most indulgent of his friends and the least charitable of his critics, and says it will give joy to both classes. Get the book and read it and get your share of the joy—whether friend or foe. Great is Lydston.

---

PREVENTION AND TREATMENT OF ABORTION. By Frederick J. Taussig, A. B., M. D., Lecturer on Gynecology, Medical Department Washington University, etc., St. Louis. Fifty-nine illustrations. St. Louis: C. V. Mosby Co. 1910. Large 8vo. 180 pages. Cloth, $2.00.

This is a timely book and will be appreciated by the general practicer of medicine who realizes that the subject is inadequately dealt with in the text-books. It is a most important branch of the physician's art, dealing with abortion, and the directions for the proper management and treatment of the pregnant woman looking to the possibility or probability of miscarriage are full, intelligent and complete. The book is replete with instructions, suggestions and facts, with minute directions as to what to do and how to do it when an abortion has occurred. The chapter on predisposing causes and exciting causes, as well as that on differential diagnosis, are especially interesting and valuable, particularly to an inexperienced obstetrician. We can and do recommend it as worth while.

